# The Relationship between Sleep and Physical Activity by Age, Race, and Gender

**DOI:** 10.31083/j.rcm2510378

**Published:** 2024-10-23

**Authors:** Taylor McCoy, Anthony J. Sochan, Andrea M. Spaeth

**Affiliations:** ^1^Department of Kinesiology and Health, Rutgers University, New Brunswick, NJ 08901, USA; ^2^Renaissance School of Medicine, Stony Brook University, Stony Brook, NY 11794, USA

**Keywords:** exercise, sex differences, circadian rhythm

## Abstract

Cardiometabolic diseases remain the leading cause of death in the United States. Lifestyle factors contribute the majority of risk for these diseases. Although diet and exercise have been the primary focus of research on modifiable behaviors to target for interventions to prevent cardiometabolic disease, recent evidence suggests that sleep also plays an important role. Indeed, the updated American Heart Association campaign includes sleep as one of its “Essential Eight”. This review details the reciprocally reinforcing positive relationship between sleep and daytime physical activity behaviors and explores how this relationship differs based on age, gender and race. For example, interventions to improve moderate intensity physical activity may be particularly beneficial to women, older adults, and Black Americans, who are at increased risk for sleep disturbances. Communicating to Americans the importance of managing their time to meet current physical activity and sleep recommendations is a challenge given that there are so many competing behaviors consuming large amounts of time (e.g., social media, gaming), but is critical given the importance of these behaviors for cardiometabolic health.

## 1. Introduction 

Cardiovascular and metabolic diseases remain leading causes of death in the United States [1] and lifestyle factors contribute the majority of the risk for these diseases [2]. The American Heart Association recently released its updated health communication campaign aimed at reducing heart disease in America by targeting 4 health behaviors (diet, physical activity, sleep and smoking), and 4 health measures (blood glucose, blood lipids, blood pressure and weight) [1]. This campaign is based on consistent, comprehensive evidence showing that consuming a healthy diet, meeting physical activity guidelines, getting sufficient high-quality sleep, and not smoking, drastically improves cardiovascular health [1, 3, 4]. It is increasingly difficult to meet these lifestyle recommendations given the continued modernization of our environment which favors calorically dense, processed foods, sedentary work and leisure activities, and limited time for sleep due to competing behaviors (social media, gaming, work, etc.) [5]. These health behaviors are also interconnected such that changes in one can impact the others [5], and this interconnection can contribute to positive (healthy diet associates with better sleep) or negative (unhealthy diet associates with poorer sleep) outcomes. This review focuses on the relationship between sleep and physical activity behaviors. Given the importance of gender, age and race differences in cardiometabolic risk [6], we will review the relationship by group.

## 2. Sleep and Physical Activity

Sleep health is multidimensional and encompasses measures of duration (total sleep time, time in bed for sleep), continuity (sleep onset latency, sleep efficiency, wakefulness after sleep onset), timing and regularity (consistent and appropriate bedtime and wake time), and subjective quality (perceptions of sleep quality/disturbances). Sleep/wake behavior (duration, timing, and continuity) is typically measured using a wrist-worn actigraph [7]. Actigraphy is widely used in the sleep field and has been validated against electroencephalogram (EEG); it shows high accuracy and sensitivity in distinguishing between sleep-wake states [8]. Perceptions of sleep (e.g., subjective sleep quality, subjective sleep duration and sleep onset latency) are measured with standardized questionnaires or survey questions. The Pittsburgh Sleep Quality Index (PSQI) is the most commonly used [9, 10]. It has been validated in clinical and community populations, among racially diverse men and women of varying ages, and shows alignment with objective sleep measures [11]. Sleep duration recommendations vary by age (see Table 1, Ref. [12]). For most adults, it is recommended that they: set aside 7–9 h/night for sleep with consistent bedtimes and wake times, exhibit a sleep onset latency of 10–20 minutes and a sleep efficiency >85%, and feel restored and alert during waking periods [13].

**Table 1.** **Sleep duration recommendations by age**.

Age	Recommended (h)
Newborns 0–3 months	14 to 17
Infants 4–11 months	12 to 15
Toddlers 1–2 years	11 to 14
Preschoolers 3–5 years	10 to 13
School-aged children 6–13 years	9 to 11
Teenagers 14–17 years	8 to 10
Young adults 18–25 years	7 to 9
Adults 26–64 years	7 to 9
Older adults ≥65 years	7 to 8

Hirshkowitz, *et al*. (2015) [12]. National Sleep Foundation’s updated sleep duration recommendations: final report *Sleep Health*;1(4):233–243. doi: 10.1016/j.sleh.2015.10.004. PMID: 29073398.

Physical activity (PA) encompasses any bodily movement produced by skeletal muscles that requires energy expenditure [14]. This includes movement related to leisure, transportation, work, and household management. Exercise is planned, intentional PA to enhance or maintain fitness and overall health; this includes balance, flexibility, strength, resistance and aerobic training [14]. Thus, PA includes exercise. According to the 2018 Physical Activity Guidelines for Americans, the recommended amount and type of PA varies by age (see Table 2). Adults should aim for 150 minutes of moderate-intensity aerobic activity (or 75 minutes of vigorous aerobic activity) and two sessions of moderate- to high-intensity muscle strength training activity each week [15]. A combination of both aerobic activity and strength training provides maximal benefits for cardiovascular and metabolic health [16]. Physical activity is typically measured via an accelerometer worn on the hip [17] or via standardized questionnaires that have been validated against objective measures of energy expenditure (i.e., International Physical Activity Questionnaire) [18].

**Table 2.** **Physical activity recommendations by age**.

Age	Recommendation
Preschoolers 3–5 years	Preschoolers should be physically active throughout the day to enhance growth and development.
	Caregivers should encourage active play that includes a variety of activity types.
Children and adolescents 6–17 years	Children should have opportunities and be encouraged to participate in physical activities that are appropriate for their age, that are enjoyable, and that offer variety.
	60 minutes (1 hour) or more of moderate-to-vigorous physical activity daily that includes:
	Moderate- to-vigorous intensity aerobic physical activity at least 3 days a week.
	Muscle-strengthening physical activity at least 3 days a week.
	Bone-strengthening physical activity on at least 3 days a week.
Adults	Move more and sit less throughout the day.
	At least 150 minutes to 300 minutes a week of moderate-intensity, or 75 minutes to 150 minutes a week of vigorous-intensity aerobic physical activity.
	Muscle-strengthening activities of moderate or greater intensity that involve all major muscle groups on 2 or more days a week.
Pregnancy and the postpartum period	At least 150 minutes of moderate-intensity aerobic activity a week.
	Women who are habitually engaged in vigorous-intensity aerobic activity or who were physically active before pregnancy can continue these activities during pregnancy and the postpartum period.
	A health care provider should monitor the progress of the pregnancy and advise women how to adjust their physical activity during pregnancy and after the baby is born as needed.
Older adults	The key guidelines for adults also apply to older adults.
	In addition, the following key guidelines are just for older adults:
	As part of their weekly physical activity, do multicomponent physical activity that includes balance training as well as aerobic and muscle-strengthening activities.
	Level of effort for physical activity should be individualized and relative to their level of fitness.
	Older adults with chronic conditions should understand whether and how their conditions affect their ability to do regular physical activity safely.
	When older adults cannot do 150 minutes of moderate-intensity aerobic activity a week because of chronic conditions, they should be as physically active as their abilities and conditions allow.

Based on https://health.gov/sites/default/files/2019-09/Physical_Activity_Guidelines_2nd_edition.pdf.

Despite the known benefits of high-quality sleep and PA for health, less than 25% of Americans report meeting the current PA guidelines [19] and less than 66% habitually obtain enough high-quality sleep [20]. Studies of tribes still living as hunter-gatherers highlight the evolutionary drive for humans to maintain high levels of PA throughout the lifespan [21]. Tribe members exhibit high quality sleep of sufficient duration that is strongly aligned with light-dark, temperature, and seasonal cycles [22]. Sleep difficulties were rare (e.g., there was no language for insomnia). As humans have modernized, it has become increasingly difficult to maintain high levels of PA [23] and maintain sleep-wake patterns that are in tune with our internal physiology and external environments [24]. Recent papers have highlighted the negative impact that urbanization has had on PA due to safety concerns, lack of green space, and reduced walkability [25] as well as on sleep due to safety concerns, light and noise pollution, and the 24/7 nature of modern society [26]. The increased use of technology and screens for work, education and leisure has also had a negative impact on PA [27] and sleep [28] due to its sedentary nature, emitted light, and potentially anxiolytic and addictive content [29, 30, 31, 32]. As humans continue to modernize, more effort will be required to meet PA and sleep recommendations, particularly for those who are socioeconomically disadvantaged and thus more likely to live in areas with greater safety concerns and noise/light pollution and less access to spaces that allow for PA and high quality sleep [33, 34].

In both men and women, higher levels of daily PA associate with better sleep outcomes [35, 36, 37, 38, 39, 40, 41]. Reciprocally, obtaining sufficient high-quality sleep at night increases the likelihood of engaging in higher levels of PA the following day [42, 43, 44, 45, 46]. In a large sample of young adults, meeting the guidelines for both aerobic activity and strength training was associated with an earlier bedtime, more positive affect, and less anxiety and anger [47, 48]. Engaging in exercise of varying types (flexibility, aerobic, strength) was also consistently associated with better sleep and fewer insomnia symptoms (i.e., difficulty falling asleep and/or staying asleep) [44, 49, 50, 51, 52, 53, 54, 55, 56]. A recent paper also demonstrated that integrating more PA through everyday activities (e.g., biking to work) improved sleep regularity (i.e., sleeping at consistent times) [57]. Higher levels of PA and exercise participation also have known benefits for circadian alignment, vasoconstriction, and brain health (e.g., increased blood flow, neurogenesis, reduced inflammation, reduced oxidative stress) which likely contribute to the observed positive effects on sleep [58, 59]. In addition, prior work has demonstrated that engaging in moderate-to-vigorous levels of PA has anxiolytic effects and associates with improved mood which likely further improves sleep, particularly by promoting a shorter sleep onset latency [60]. Collectively, evidence supports current clinical, World Health Organization (WHO) and Centers for Disease Control and Prevention (CDC) recommendations to increase PA in order to improve sleep [61, 62].

Timing and intensity of PA are important factors to consider. In terms of intensity, there may be an inverse-U relationship such that very high levels of PA/excessive exercise negatively impact sleep including difficulty falling asleep and increased sleep disruption [63, 64]. The timing of PA, particularly high intensity PA, may partially contribute to this observation. Some studies as [65] have observed that late-night exercise (e.g., exercise within an hour of bedtime) results in sleep-inhibiting conditions (sustained increase in body temperature (1.5 °C–2.5 °C), increased heart rate, and greater sympathetic nervous system activation) and subsequent impairments in sleep. In a large sample of healthy adults recruited from campus recreation centers, we measured sleep using the PSQI and found that individuals exercising later in the evening reported later bedtime, longer sleep latency, lower sleep efficiency, poorer sleep quality, and a higher global PSQI score [47]. However, other studies have not observed an adverse effect of evening exercise, and an individual’s fitness level and/or chronotype may moderate these relationships [66, 67, 68]. Chronotype reflects an individual’s preference for sleep/wake timing with some preferring morning activity and an earlier bedtime, and others preferring evening activity and a later bedtime [69]. In the aforementioned study on exercise timing and sleep in young adults, we observed that morning chronotypes were more likely to exercise in the morning and evening chronotypes were more likely to exercise in the evening and that the evening chronotype was also independently predictive of poor sleep outcomes [47]. There are well known associations between evening chronotypes and adverse mental and physical health outcomes [70], including greater sedentary behavior [70]. Thus, it is important to measure chronotype in studies focusing on exercise timing to reduce confounding effects. In conclusion, increasing daily PA levels to meet current guidelines and incorporating moderate-intensity exercise into an individual’s daytime routine, particularly before nighttime hours, will likely positively impact sleep.

Reciprocally, studies have consistently demonstrated that children, adolescents and adults who report sufficient sleep duration exhibit better physical fitness, engage in more PA and are more likely to meet current PA guidelines [44, 49, 50, 71, 72, 73, 74, 75, 76, 77]. Those with better sleep (increased sleep efficiency, shortened sleep onset latency, fewer awakenings after sleep onset, and more deep sleep) also exhibited greater exercise exertion and higher PA levels [78, 79]. Individuals who were sleep restricted were less likely to engage in moderate-to-vigorous intensity PA and exhibited impaired muscle strength the following day [80]. Sleep debt may promote muscle degradation pathways, impair recovery, and increase fatigue which would make it more difficult to sustain routine PA [81]. Sleep loss also decreased motivation and increased negative mood which could lower the desire to exercise [82]. Thus, these two health behaviors appear to be mutually reinforcing and may have important interactive effects for brain [83] and cardiovascular health [84]. It is critical for societal systems to promote PA and sleep given the increased effort that needs to be devoted to these health activities due to modernization.

## 3. Gender Differences

It is important to consider the relationship between PA and sleep separately for men and women. Compared to men, women have been more likely to report sleep disturbances [85], exhibit insufficient PA and exercise for different reasons [86, 87] and these differences emerge around puberty [88, 89]. In addition, studies have observed gender differences in the relationship between sleep and exercise [62, 90, 91]. For example, a meta-analysis found that the benefits of acute exercise on objective measures of sleep (i.e., for non–rapid eye movement stage 1 sleep duration and wake time after sleep onset) were stronger for men than for women [62, 92, 93]. Other studies have shown that engaging in morning exercise outdoors positively correlates with better sleep quality and a longer nocturnal sleep duration in women only [94] and that sufficient sleep predicts better cardiorespiratory fitness in adolescent girls, but not boys [95]. In a large observational study, women reported poorer sleep quality than men but the threshold for PA improving sleep quality was lower in women (600–6000 METs (metabolic equivalents)-min/week) than men (6000–9000 METs-min/week) [96].

In our study of young adult exercisers, we found that women reported fewer exercise sessions per week and poorer sleep efficiency compared to men, and that exercise was associated with different sleep outcomes in men and women [48]. Men who met PA guidelines reported taking less time to fall asleep than those who did not. Difficulty falling asleep is a hallmark of insomnia. Given that men are less likely to seek treatment for insomnia compared to women [97], “exercise is medicine” for insomnia may be an important treatment option as it could be perceived as more acceptable to men than traditional therapies (e.g., CBT-I, cognitive behavioral therapy for insomnia). Women who met the PA guidelines went to bed earlier than those who did not. In women each additional exercise session was associated with going to bed 13 minutes earlier and this is an important finding given that women were more likely to report time burden as a main reason for not exercising as often as they used to [48]. Time limitations related to work, commute, and social obligations in a 24-hour day often result in reallocation of time dedicated to exercise and sleep [98] and yet in our study women who exercised more were able to prioritize an earlier bedtime. Other studies have shown that maintaining an earlier bedtime is associated with improved productivity which may explain how these women were able to make time for exercise [99, 100].

Women also experience periods of marked hormonal fluctuations during the menstrual cycle, pregnancy and menopause that may influence both health behaviors and their interactions [93, 101, 102]. For example, women have reported better sleep and an increased likelihood of participating in PA during certain menstrual phases compared to others [93] and women who were on hormonal contraception exhibited better sleep and higher levels of PA compared to women who were not [103]. There is also an interesting disconnect in women such that they exhibit worse subjective measures of sleep quality (i.e., self-report questionnaire) [85, 90, 104, 105] but better objective measures of sleep quality (i.e., sleep efficiency assessed using overnight polysomnography) [106] compared with men [104]. Exercise interventions have been shown to improve subjective and objective measures of sleep in women, particularly as they undergo menopause [107]. A study that implemented a six-week, twice/week exercise intervention in females with generalized anxiety disorder found that sleep onset latency decreased post-intervention for women engaging in resistance training or aerobic exercise [108]. Pregnancy is often associated with poor sleep and decreased PA [109] and a recent meta-analysis found that exercise interventions during pregnancy increase PA, reduce sleep disturbances and improve sleep quality [110]. Sleep issues are one of the most common complaints of women transitioning through menopause and a recent study implementing moderate-intensity exercise or stretching in the morning for one year observed improvements in sleep latency, sleep quality, use of sleep medications, physical fitness, and time spent outdoors in menopausal women, with significant correlations observed between improvements in fitness and sleep [111]. Similarly, another study observed improvements in sleep for menopausal women in a resistance training intervention and found a negative correlation between plasma estradiol levels and insomnia symptoms post-exercise-intervention, again suggesting an important contribution of hormones to the relationship between PA and sleep [112]. More studies are needed to examine how PA and exercise affect subjective and objective sleep measures in women and these studies should be sure to collect information about the menstrual cycle and use of contraception. Similarly, testosterone levels are influenced by sleep [113] and are predictive of PA levels [114] in men so it is important to consider reproductive hormones in both genders.

Given the importance of sleep and PA as health behaviors for the prevention of chronic diseases, and the complexities of gender differences in physiology and behavior, more work is needed in this area. Interestingly, the relationship between sleep and coronary artery calcium (CAC), a major indicator of atherosclerosis, varied by gender such that insomnia symptoms correlated with poorer CAC scores in women and not men [115]. In this study, PA levels were significantly higher in men than in women. Thus, it is possible that PA served as protective against the effects of short sleep on CAC in men. Future work is needed to better understand the relationship that sleep and PA share with cardiovascular biomarkers.

## 4. Age

Sleep and PA behaviors vary markedly across the lifespan. For example, children are highly physically active (and indeed, have a hard time staying sedentary) and require long sleep durations that have higher proportions of rapid eye movement (REM) and slow wave sleep [72]. After puberty, there is an increase in the reporting of sleep disturbances and PA levels drop dramatically [72, 116, 117]. In adulthood, many individuals find it difficult to manage time and habitually maintain sufficient sleep and PA due to work and family obligations [118]. Finally, in older adulthood, there is more time for sleep and PA due to retirement but the ability to engage in these health behaviors may be limited due to other health issues (e.g., pain, frequent urination, frailty, chronic diseases) [118]. Thus, it is important to understand the relationship between PA and sleep across the lifespan [91].

Evidence has suggested that children exhibit a positive relationship between PA and sleep quality/duration; however, there is debate as to the strength of this relationship due to high heterogeneity of research [46, 119]. Children as young as 1 to 3 years who engaged in higher levels of PA, particularly when it was performed outside, exhibited shorter sleep onset latencies, better sleep quality and more consistent sleep patterns [120]. Outdoor PA has also been associated with shorter sleep onset latency, and longer sleep duration and sports participation has been related to better sleep quality (efficiency and duration) and earlier bedtimes in children [120, 121, 122]. Similarly, school-aged children exhibited increased sedentary behaviors and decreased PA levels after a short sleep duration [123]. However, a few studies as [124] have shown that sleep may be impaired by PA of higher intensity and longer duration. Overall, if given the opportunity for sufficient sleep and PA (e.g., parents provide time and space for these behaviors), most children do not have issues with their ability to engage in high levels of PA and sleep soundly.

Adolescents are an important age group to target as this is when sleep issues emerge, PA levels decrease, and certain cardiometabolic risk factors can become apparent (e.g., obesity) [125]. Time use and chronotype are also critical factors at this age. School start times, high homework loads, social media use and extracurricular activities impede on the time available for sleep and exercise [126]. After puberty there is also a shift towards evening chronotype such that adolescents prefer to be active later in the day and have a delayed sleep period (later bedtime and waketime) [126]. This shift combined with early school start times makes it difficult for teens to obtain sufficient sleep even if their ability to sleep is not impaired [126]. Findings regarding the relationship between engagement in moderate-to-vigorous PA and sleep duration have been somewhat mixed, possibly due to these factors [127, 128]. In adolescents with obesity, increasing PA level (steps per day and average daily METs) improved sleep duration and sleep efficiency [129]. Another study demonstrated that twelve weeks of exercise training decreased non-REM stage 1 sleep and fragmentation, and increased REM sleep and sleep efficiency [125].

During young adulthood, particularly for those enrolled in college, PA levels tend to be higher and sleep duration decreases [72]; however, there are dynamic changes across the semester [130]. Social activities and studying for exams can negatively influence both sleep and PA levels [130] and although it is typical for universities to implement amenities to facilitate PA (e.g., campus recreation facilities, intramural sports), insufficient sleep is embedded into the culture of college life (e.g., all-nighter events during exams, libraries open 24/7). The relationship between PA and sleep is mutually reinforcing such that increased PA associates with improved sleep [131, 132, 133] and both behaviors associate with better quality of life [134]. However, as stated above, exercise timing seems to be important, with more positive effects (improved subjective sleep quality, reduced sleep latency, reduced wake after sleep onset) associated with morning/afternoon exercise compared to evening exercise [133, 135]. Given recent surges in mental health issues among teens and young adults, it is becoming increasingly important for university student health services to address sleep and PA behaviors and implement positive health interventions [39, 62, 136, 137]. Even in our large, diverse sample of college students who were recruited from campus recreation centers, only 47% of students reported meeting current PA guidelines, 56% reported poor sleep quality, 37% report habitual insufficient sleep (<7 hours/night), and mood disturbances were common (41% of students reported moderate to severe levels of anger, anxiety, or depression) [47, 48]. Meeting PA guidelines and exercising more days per week associated with significant improvements in sleep and mood [47, 48]. University intervention programs, conducted by kinesiology and student health departments, are needed and have begun to be initiated by the national “Exercise is Medicine on Campus” initiative (i.e., Temple University College of Public Health) [138, 139]. These interventions provide one-on-one exercise prescriptions to students who visit campus health facilities, offer first-year seminars about exercise resources on campus and the importance of exercise, integrate health technology into campus infrastructure to monitor patient progress, and host outreach events to promote exercise in [138, 139]. Similar programs have been piloted related to sleep (i.e., Healthy Sleep program at Harvard University), but the movement is in its infancy.

As adults age, the time for sleep and daily PA is impeded by work and family obligations and the ability to engage in these health behaviors may start to decrease due to other issues. After retirement, the issue is less about limited opportunities/time for these behaviors and is more related to the *ability* to engage in PA and sleep of sufficient duration and quality. In middle aged adults, increased physical fitness, higher PA and greater muscular strength are associated with better subjective sleep quality and duration [45, 140, 141] and an exercise intervention that increased daily step count significantly improved subjective sleep quality [118]. These findings are consistent with a recent meta-analysis that found exercise was associated with better sleep, with stronger effects on subjective measures of sleep quality than on objective measures of sleep latency, duration or architecture [62]. Interestingly, one study differentiated the basis of PA in middle-aged adults and found that higher leisure-time PA, but not occupational PA, was associated with better sleep [94, 113]. Thus, for middle-aged adults, setting aside time for planned leisure PA may be particularly important for subsequent mental health and sleep outcomes.

Decreased PA and lean mass, and increased risk for frailty, balance issues and falls, are hallmarks of aging. Implementing exercise programs that incorporate both aerobic and resistance training is critical in the aging population to reduce these adverse outcomes. Further, aging is the strongest risk factor for heart disease, cancer neurodegenerative diseases and other leading causes of death. Sleep and PA are known to be important contributors to these diseases [60]. Issues with the ability to fall asleep and stay asleep (e.g., insomnia) are common in older age groups and evidence suggests that older individuals who remain physically active, physically fit, and maintain higher levels of lean mass, exhibit better sleep outcomes [56]. Exercise as a treatment for insomnia in older adults has been vetted as a safe and feasible treatment that should be further explored and applied [142]. In an observational study of older adults, those who met recommended moderate and vigorous PA guidelines reported better subjective sleep quality compared to those who did not and those who participated in leisure walking exhibited better sleep duration compared to those who did not [143], suggesting that even incremental increases in PA are beneficial. Mind-body programs (e.g., Tai Chi) may be particularly beneficial for improving sleep in older adults as they address anxiety and mood more directly [144]. However, other more traditional exercise programs have also had positive impacts on mood and sleep. For example, a 10-week randomized controlled trial in a sample of older adults found that a supervised weight-training program three times a week significantly improved subjective sleep-quality, quality of life, and depression symptoms compared to an attention-control group [145]. Thus, the benefits of PA for sleep are particularly pronounced in older adulthood and the two behaviors remain mutually beneficial.

Both PA and sleep are particularly critical for maintaining brain and cardiovascular health and reducing the risk of dementia and neurodegenerative diseases [146] and cardiometabolic diseases [3, 83, 147]. Although the relationships between sleep, PA and cardiometabolic risk becomes more imminent in older adulthood, these relationships are evident throughout the lifespan [148]. Children who obtain sufficient sleep and/or have fewer sleep problems exhibit better insulin sensitivity and glycemic control, lower adiposity, and are at decreased risk for type 2 diabetes (T2D) and dyslipidemia compared to children who do not [149, 150, 151, 152]. Similarly, children who are more physically active also exhibit better glucose regulation and are at lower risk for type 2 diabetes compared to children who are sedentary [153, 154]. Vigorous intensity exercise that is high-impact and weight-bearing is particularly important for children as this training promotes bone, cardiovascular and metabolic health [155]. Adolescents with longer sleep duration and earlier bedtimes exhibit lower body mass index (BMI) across genders, as well as smaller waist circumference in boys [151]. Lower PA levels and not participating in sports are also risk factors for obesity, as measured with BMI [156]. Interestingly, a study by Del Pozo-Cruz *et al*. [157] found that replacing sedentary behaviors with either PA or sleep resulted in decreased BMI among young children and adolescents. In a sample of young adults, PA but not sleep quality was significantly associated with serum inflammatory measures, insulin, leptin, and triglyceride [134]. In adults, insufficient sleep duration and low levels of PA have been associated with high blood pressure, insulin resistance and high blood glucose levels, altered cholesterol transport and metabolism, and increased adiposity and inflammation [1]. Collectively, this evidence supports public health efforts to improve sleep and PA across the lifespan to prevent cardiometabolic diseases, which contribute to 18 million deaths per year in America (as of 2019 according to the WHO).

## 5. Race

Consistent with broader systemic health disparities in America [158, 159], Black individuals in the United States are more vulnerable to sleep health disparities [160]. Several studies have shown that Black children as young as 2 years are more likely to habitually obtain insufficient sleep of poorer quality compared to their white counterparts [161] and this disparity continues through adolescence [161] and into adulthood [162]. Black individuals are also more likely to exhibit longer sleep onset latency, napping, and poorer sleep efficiency compared to white individuals [163, 164, 165, 166, 167]. In controlled settings, some race differences in physiological sleep persist (decreases in slow wave sleep, increases in non-REM stage 1 and 2 sleep) [168, 169, 170] and may be associated with ancestral genes [171], however, it is believed that the majority of these differences are driven by social determinants, including socioeconomic status [172] discrimination [173] and neighborhood safety [174]. Racial disparities in PA also emerge during adolescence, when PA significantly decreases [175], and persists into adulthood [176, 177, 178]. These differences are driven by the same social determinants as sleep – socioeconomic status [179], discrimination [180] and neighborhood safety [181]. In addition, structural inequities in the access to community green spaces that facilitate PA is rampant in the United States and drives low PA levels in underserved communities [182, 183], due to historical redlining practices and other forms of housing discrimination [184, 185]. Interventions to increase PA have shown positive results, with Black individuals responding more strongly than white individuals in some studies as [186]. It is also important to note that on a global scale, lower income nations actually show higher levels of PA as occupations and commuting options for these individuals require high levels of PA [187, 188]. Thus, race differences in sleep and PA may be unique to the United States and/or higher gross domestic product (GDP) nations.

There is a paucity of research examining race differences in the relationship between PA and sleep; however, an interesting paper in adolescents found that at higher levels of PA, sleep duration was sufficient and did not differ by race whereas at lower levels of PA, Black adolescents exhibited a significantly shorter sleep duration [189]. This suggests that interventions aimed at increasing PA in youth may serve as a protective factor against racial disparities in sleep. In adults, one study found no difference between Black and white individuals in terms of the relationship between PA and sleep [190]. Another highlighted the importance of PA type such that there was a protective effect of leisure PA in white individuals only whereas in Black individuals, greater occupational PA was actually associated with a shorter sleep duration [3, 191]. Indeed, while white adults are more likely to engage in leisure PA, Black adults are more likely to engage in occupational PA [192] thus, it is important to collect information related to the type/context of PA when examining the relationship between PA and sleep.

Racial disparities and social determinants of health are critically important to understanding the prevention and treatment of cardiometabolic diseases. In the Essential Eight campaign, the American Heart Association highlights the need for more work in this area and acknowledges that sleep health disparities may play an important role in understanding the racial differences in heart disease [1]. Indeed, Curtis *et al*. [193] found that differences in sleep between Black and white individuals explained 41% (sleep duration) and 58% (sleep efficiency) of the racial differences in cardiometabolic disease risk. Black adults may be more vulnerable to weight gain when sleep was restricted compared to white adults [194, 195, 196] and exhibit different metabolic responses to sleep restriction [196]. The relationship between low PA levels and increased cardiometabolic disease risk may be particularly evident in Black women, who are at the highest risk for low PA levels and obesity [197]. In a sample of racial minority socioeconomically disadvantaged women during the postpartum period, it was observed that short sleep predicted greater weight gain for women with obesity and that fewer self-reported minutes of walking was associated with a decreased sleep duration [196, 198]. However, the ability of the researchers to examine the relationship between PA and sleep duration was limited to only “minutes spent walking” due to the extremely low levels of PA reported by this population. Future research is needed to examine the relationship between sleep and PA in Black women, and the postpartum period may be an important time window to target. A six-month community-based intervention aimed at increasing PA in women effectively reduced cardiometabolic disease risk comparably in Black and white women [199], suggesting that devoting more resources to targeting PA could have a large impact on the prevention of cardiometabolic disease in this group.

A recent comprehensive review of sleep health disparities highlighted recent interventions targeting sleep in the racial minority communities [200]. For example, a yoga + sleep hygiene education intervention delivered to individuals living in an affordable housing community led to increased sleep duration (5.4 hours/night to 6.9 hours/night) and reduced sleep disturbances [201]. It is critical that these interpersonal interventions factor in stress and trauma to address issues of discrimination, racism, and poverty that represent barriers to sleep health in the Black community. At the policy level, the authors highlight the importance of eliminating systemic racism, increasing the diversity of neighborhoods, and improving the built environment to reduce sleep disparities. They note that creating green spaces to encourage PA, improve air quality, enhance neighborhood connections, and reduce noise and light pollution are critical to addressing health disparities, including sleep [62, 202, 203, 204, 205]. Thus, interventions targeting PA and sleep using racially informed approaches have the potential to prevent cardiometabolic diseases and reduce health disparities.

## 6. Conclusions

Sleep and PA, particularly PA of moderate intensity carried out during daytime hours, share a reciprocally reinforcing positive relationship that promotes cardiometabolic health. Interventions to improve PA may be particularly beneficial to women, older adults, and Black Americans, who are at increased risk of sleep disturbances. It is critical that future studies focus on the implementation of interventions, at the level of the individual as well as policy, to effectively improve these health behaviors. Targeting adolescence, a time when PA levels decrease and sleep disturbances emerge, may be a critical period to help individuals develop healthy habits and prevent disease. More work is also needed to tailor these interventions for socioeconomically disadvantaged individuals, particularly those who are experiencing chronic stress due to discrimination and racism. Few trials have developed culturally sensitive interventions targeting sleep and PA. In addition, communicating to Americans the importance of managing their time to meet current PA and sleep recommendations is a challenge given that there are so many competing behaviors consuming large amounts of time (e.g., social media, gaming). Effective health communication strategies are needed to raise awareness about the potential health consequences of devoting so much time to screens/technology at the expense of sleep and PA. Finally, we acknowledge that this review focused on sleep/wake behaviors rather than the presence of sleep disorders such as obstructive sleep apnea. Obstructive sleep apnea is associated with low PA levels and poor physical fitness and increases the risk of cardiometabolic diseases [55]. Future reviews should focus on understanding the relationship between obstructive sleep apnea, PA and cardiometabolic disease by gender age and race.
